# The Conventions

**Published:** 1890-07

**Authors:** James Truman


### THE CONVENTIONS.

BY the time this number reaches its readers, the members of the profession, both in this country and in Europe, will be preparing for the annual gatherings. This year is peculiarly full in this direction and promises to be one prolific in changes; whether for good or ill will depend on those who have the controlling influence. The need of readjustment of old lines of labor must be quite apparent to the average observer. The present status of convention work is not up to the level of its origin. The defects are apparent to all; but the remedy is not so clear. There is one point that all the active workers will agree upon, and that is that the profession, as a whole, fails to appreciate the importance of maintaining, to the highest standard, our representative associations, whether they be held in this country or Europe. The work of supporting these falls upon a fraction of the twenty thousand dentists, probably, now in the United States. It would seem that, aside from the natural desire to meet with and hold fraternal communion with widely separated members of a common calling, there should be a feeling, rising to an inspiration of duty, calling all to assemble at least once a year and aid by their presence, if not by more active effort, the work of progressive development.

This year the American Dental Association will meet at Excelsior Springs, Mo. It will open up to many in the East a favorable opportunity, not only to meet our Western brethren, but to make the acquaintance of a most interesting portion of our country under the most favorable circumstances. That the dentists of that section will be there in force is assured; but it will be a matter of regret if other portions fail to respond as they should.

The International Medical Congress of Berlin will, doubtless, draw a large number of the old and earnest workers away, and it is to be regretted that an effort was not made last year to avoid a clashing of time, for, as the matter stands, it will have a tendency to weaken the representation in both directions,—Excelsior and Berlin. An earnest effort has been made to correct this mistake by calling the American Dental Association at an earlier period; but up to this writing there has been no change made in the original programme.

The work of all these bodies will soon be laid before the readers of the professional journals, and should prove an incentive to greater individual and collective work in the future.

James Truman.

